# Cross-sectional analysis of W-cored Ni nanoparticle via focused ion beam milling with impregnation

**DOI:** 10.1186/1556-276X-9-533

**Published:** 2014-09-27

**Authors:** Saeeun Jeong, Hyunwoong Na, Gwangyeob Lee, Seong Ho Son, Hanshin Choi

**Affiliations:** 1 Incheon Regional Division, Korea Institute of Industrial Technology, Incheon, 406-840, Republic of Korea; 2 Department of Materials Science and Engineering, Yonsei University, Seoul, 120-749, Republic of Korea

**Keywords:** W-Ni bimetallic nanoparticle, RF thermal plasma, Nonequilibrium phase, Nanoparticle cross section, TEM

## Abstract

Tungsten and nickel bimetallic nanoparticle is synthesized by radio frequency thermal plasma process which belongs to the vapor phase condensation technology. The morphology and chemical composition of the synthesized particle were investigated using the conventional nanoparticle transmission electron microscopy (TEM) sample. A few part of them looked like core/shell structured particle, but ambiguities were caused by either TEM sample preparation or TEM analysis. In order to clarify whether a core/shell structure is developed for the particle, various methodologies were tried to prepare a cross-sectional TEM sample. Focused ion beam (FIB) milling was conducted for cold-compacted particles, dispersed particles on silicon wafer, and impregnated particles with epoxy which is compatible with electron beam. A sound cross-sectional sample was just obtained from cyanoacrylate impregnation and FIB milling procedure. A tungsten-cored nickel shell structure was precisely confirmed with aid of cross-sectional sample preparation method.

## Background

Multi-layer ceramic capacitor (MLCC) is composed of a ceramic dielectric layer and a metallic electrode layer. The replacement of precious electrode material with nickel electrode plays a pivotal role on exploiting MLCC in electronic components [1,2]. Miniaturization with higher performance has been persistently required and it is achieved by reducing the thickness of both dielectric layer and electrode layer. When it comes to the MLCC fabrication, conductive ink containing nickel nanoparticles is overlaid on dielectric green sheet by screen printing process and then it is stacked and sintered simultaneously. In this regard, thinner layer thickness is obtained by reducing the nickel particle size [3,4].

During sintering, the sintering behavior of the metallic layer is different from the ceramic layer and it causes to form defective microstructures and deteriorate MLCC performance. The discrepancy in the sintering temperature is further enhanced with nickel by reducing the particle size. As a result, it is a critical challenge to satisfy the capacitance density with mitigation of size effects on sintering [5-7]. As a matter of fact, composite materials with addition of BaTiO_3_ or TiO_2_ nanoparticles are introduced to retard the sintering of metallic components and it is proven that the sintering temperature difference with the dielectric layer is reduced [8,9]. However, full exploitation of the electrical conductivity is not achieved by the ceramic-phase dispersed electrode layer even though the discontinuity is markedly reduced [10]. In this regard, nickel and tungsten binary systems are designed to satisfy both retarded sintering and improved electrical conductivity. Nickel has a limited solubility in tungsten while tungsten has no solubility in nickel. Also, tungsten has a slow diffusivity in nickel. Accordingly, diffusion-dependent sintering is kinematically retarded by either lower diffusivity of tungsten or tungsten particle dispersion.

Nanoparticles can be synthesized by different technologies [11-15], and precise characterization methodologies have seen developed to characterize nanoparticle properties such as structural and microstructure and chemical composition [16-19]. Moreover, these nanoparticle properties are used to understand synthetic process. Transmission electron microscopy (TEM) is a representative basis in nanoscale characterization technology [20-22]. Generally, it is facile to prepare TEM sample in the case of nanoparticle, thanks to the dimension as thin as electron transparency. Furthermore, information on nanoparticles can be accurately obtained if nanoparticles are mono-dispersed. However, nanoparticles are agglomerated during either synthesis process or sample preparation process in most cases [23,24]. It causes ambiguities and it even leads to misinterpretation with respect to nanoparticle and synthetic technology. Accordingly, the TEM sample preparation methodology needs to be further exploited with and without destructive methods. In the present study, W and Ni bimetallic nanoparticle is synthesized by reactive RF thermal plasma process which belongs to the vapor phase condensation technology [25-27]. Nanoparticles are nucleated via condensation reaction during rapid quenching of gaseous species and then they undergo collision and coalescence during flight [28,29]. Nucleation and growth reactions are deduced from the characteristics of the synthesized nanoparticle and therefore accurate characterization is crucial. RF thermal plasma-synthesized nanoparticles form agglomerates and/or aggregates like other vapor phase-condensed nanoparticles [30]. Accordingly, cross-sectional analysis with destructive thinning procedure as well as plan-view analysis with conventional nondestructive method is required to clarify the possible ambiguities in TEM analysis. To do this, a cross-sectional TEM sample for nanoparticle is explored and the impregnation and focused ion beam (FIB) milling method is suggested in this study.

## Methods

### Materials

W-Ni bimetallic nanoparticle was synthesized by feeding tungsten trioxide (WO_3_) and nickel hydroxide (Ni(OH)_2_) blended micropowder into argon-hydrogen thermal plasma at the weight fraction of 0.5. The mass flow of hydrogen gas is five times higher than the stoichiometric one which is required for full reduction of the feedstock powder. The chemical composition of the synthesized W-Ni particle was measured by scanning electron microscopy with energy dispersion spectroscopy. Tungsten weight fraction was 0.588 by areal quantification at the magnification power of 500 and it is well consistent to the theoretical value (0.556).

### TEM sample preparation

TEM samples were prepared by both conventional method and FIB milling. The nanoparticle was dispersed in ethyl alcohol, and the suspension was dropped on copper grid for the former sample preparation. On the other hand, nanoparticles were cold compacted in a rigid die mold at the normal pressure of 600 MPa and then the cross-sectional TEM sample was prepared by site-specific FIB milling. Additionally, the cross-sectional one was made by simple dispersion of the nanoparticle on silicon wafer and also by impregnation and FIB milling. In the case of the impregnation and FIB milling method, cyanoacrylate epoxy was used to mold the nanoparticles. The cross-sectional lamella is prepared by *in situ* lift-out FIB method. Additional ion milling and plasma cleaning followed. TEM and scanning transmission electron microscopy (STEM) with energy dispersion spectroscopy were conducted for plan-view sample and cross-sectional samples.

## Results and discussion

**Figure 1:**
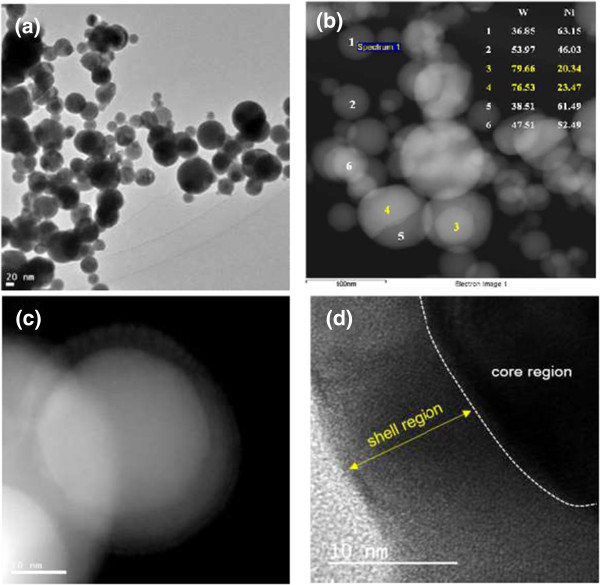
**Plan-view morphology of the W-Ni bimetallic nanoparticle by way of conventional sample preparation method. (a)** TEM image of W-Ni bimetallic nanoparticles. **(b)** STEM photograph and the chemical composition were measured by point quantification. **(c)** Core/shell structured nanoparticle. **(d)** High-resolution TEM image of **(c)**.

**Figure 2:**
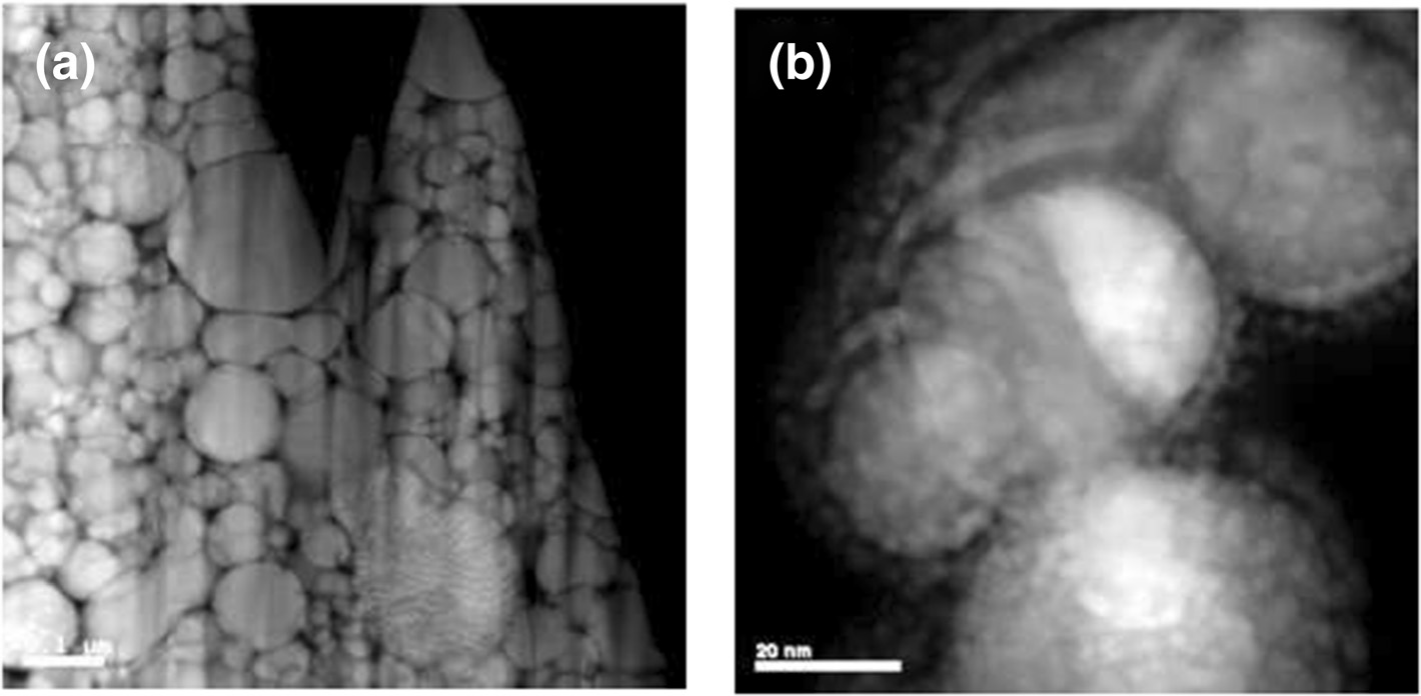
**Artifacts of the cross-sectional TEM sample via cold compaction and FIB milling. (a)** Preferential milling. **(b)** Re-deposition of sputtered species on the nanoparticle surface during milling of the dispersed nanoparticle on silicon wafer.

**Figure 3:**
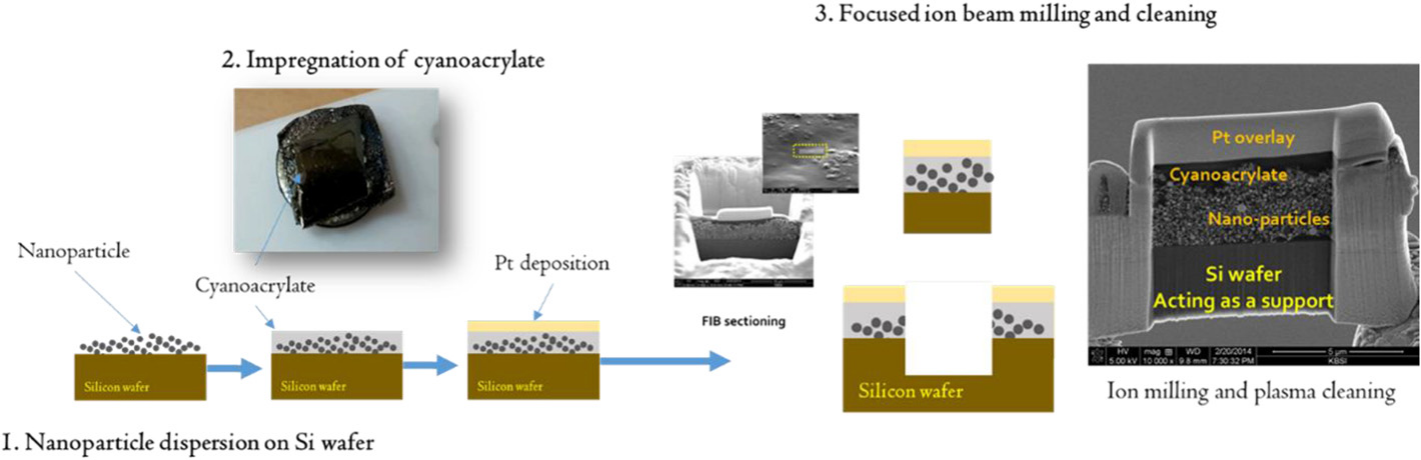
Cross-sectional TEM sample preparation via impregnation and FIB milling of the W-Ni bimetallic nanoparticle.

The plan-view characteristics of the W-Ni bimetallic nanoparticle by reactive RF thermal plasma synthesis are shown in Figure 1. W-Ni nanoparticles are spherical, and parts of them make aggregates by sintering neck formation. The chemical composition of nanoparticles at the particle level was extensively measured by point quantification in STEM-EDS analysis. Figure 1b shows an STEM image and chemical compositions of typical nanoparticles. There is a wide range of chemical composition; however, pure tungsten and pure nickel were hardly identified. The contrast with elemental quantification reveals that most particles are alloyed and some coagulated particles are observed. A spherical particle which consists of tungsten-rich part (no 4) and nickel-rich part (no 5) of Figure 1b is a good example. The morphology of the coagulated particle is not fully described in the plan-view analysis. A particle looks like core/shell structured particle as shown in Figure 1c; however, it is not clear whether the bright contrast at the central region is a cored particle or an attached particle. Even for the HR-TEM analysis, it is not solved.The cross-sectional TEM sample which was prepared from cold compaction and FIB milling is shown in Figure 2a. During FIB milling, a high-energy ion is directly irradiated for compaction and artifacts can be generated. Figure 2a shows severe fringes on the cross-sectional sample owing to preferential milling. Meanwhile, Figure 2b shows re-deposition of sputtered species on the nanoparticle surface during milling of the dispersed nanoparticle on silicon wafer. Re-deposited layer hinders examination of the structure and chemical composition. Both defects are not healed by plasma cleaning.Figure 3 shows the procedure for cross-sectional TEM sample preparation by the impregnation and FIB milling process. Firstly, W-Ni nanoparticles are dispersed on the silicon wafer. After that, cyanoacrylate is impregnated into the nanoparticle deposit. Cyanoacrylate is a kind of adhesive which is compatible to silicon wafer and accordingly, nanoparticles are embedded in the cyanoacrylate adhesive which adheres strongly with Si support. Cyanoacrylate is polymerized when it is exposed to moisture in ambient environment. Pt deposition and ion sectioning followed. Finally, the sample is cleaned by plasma surface treatment. A sound TEM sample is prepared by the procedure as shown in Figure 3.

**Figure 4:**
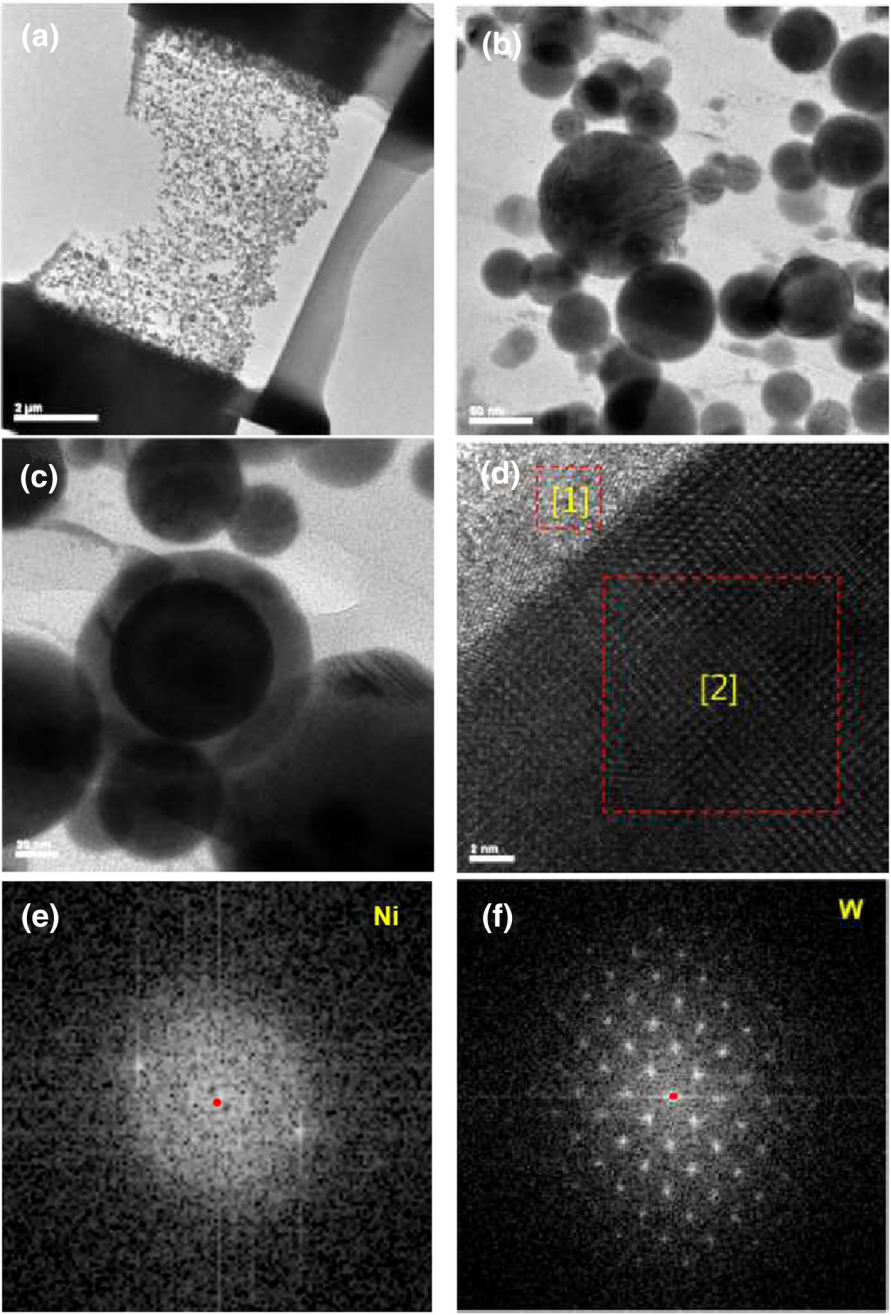
**Cross-sectional morphology of the W-Ni bimetallic nanoparticle. (a)** TEM sample from impregnation and milling. **(b)** Cross-sectional TEM photograph. **(c)** Core/shell structured nanoparticle. **(d)** HRTEM image of core/shell nanoparticle. **(e)** FFT pattern of (1) in **(d)** and **(f)** FFT pattern of (2) in **(d)**.

**Figure 5:**
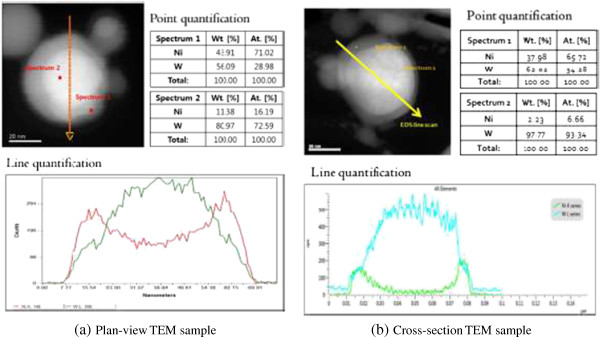
Comparison of core/shell nanoparticle’s chemical composition using conventional plan-view sample and impregnation and FIB-milled sample.

The cross-sectional TEM sample from the impregnation and FIB milling is shown in Figure 4a, and particle morphologies can be seen in Figure 4b. Firstly, nanoparticles are more uniformly dispersed when they are compared to the conventional sample. When tungsten is added to nickel, magnetic properties are highly suppressed; however, they are easily agglomerated during the conventional sample preparation owing to size-dependent particle interactions. It is the first advantage of the impregnation of cyanoacrylate. In addition, ionic milling enlarges particle numbers which are thin for electron transmission and also ambiguity of core/shell structured nanoparticle in the conventional TEM sample are clarified as shown in Figure 4c,d,e,f. The core/shell nanoparticle in Figure 4c is thick for electron transmission but it is milled so that high-resolution TEM analysis can be conducted as shown in Figure 4d. Fast Fourier transform (FFT) patterns are obtained from the core region and periphery region as shown in Figure 4e,f, respectively. In general, amorphization of binary metallic systems occurs by either rapid quenching or mechanical alloying. In the present study, the nanoparticle which has a favorable dimension in view of heat transfer is rapidly condensed and solidified. In the case of W-Ni binary system, it has a small negative enthalpy of mixing but solid-state amorphization is frequently observed in mechanical alloying when the tungsten content is above a critical content. For example, the critical tungsten content is 28 at.% in the literature [31]. The Miedema model, a semi-empirical assessment of thermodynamics, is used to calculate the enthalpy of mixing and to estimate the stability of amorphous phase. When Ni concentration in W matrix increases, the excess solute concentration increases the energy of the crystalline and therefore the amorphous phase becomes stable. In the case of MA process, increases of solute concentration and defects concentration by plastic deformation and resulting inter-diffusion result in strain energy of the crystalline. Meanwhile, the outward diffusion of Ni increases the solute concentration near the surface region where amorphization occurs partially near Ni-supersaturated shell. Tungsten-cored W-Ni shell is directly observed by the cross-sectional TEM sample and furthermore, amorphous phase is clearly identified.The presence of W core/Ni shell nanoparticle is clearly identified by the cross-sectional TEM sample preparation. The chemical composition of the core/shell nanoparticle in the cross-section image is compared to that of the plan-view particle as shown in Figure 5. In the case of plan-view TEM sample from the conventional sample preparation method, it shows a typical elemental distribution across the particle. However, the nickel content at the core is still high and accordingly, it is ambiguous whether the tungsten-rich region is cored or attached on the surface. On the contrary, the depletion of nickel is apparent for the cross section of the TEM sample and a diffusive elemental distribution of nickel to the surface is observed. More precisely, the outer shell is a W-Ni alloy. It is another advantage of the cross-sectional TEM sample preparation that clarifies the ambiguity of the plan-view TEM analysis.

When the condensation reaction from Ni and W gaseous mixture is considered, it is rational that the core/shell structured particle comes from the co-condensation of W-Ni and outward diffusion of Ni to the surface during cooling rather than heterogeneous condensation of Ni on the surface of previously condensed tungsten nanoparticle. From the all the results such as alloy nanoparticle, coagulated one, and tungsten-cored W-Ni shell nanoparticle in the as-synthesized particles, W and Ni condense simultaneously owing to rapid quenching, although the equilibrium saturation temperature of W is much higher than Ni. If the tungsten content is high, Ni diffuses to the surface because it has no solubility in W and partial amorphization of Ni-enriched shell occurs due to chemical composition-dependent stability [32].

## Conclusions

Tungsten and nickel bimetallic nanoparticle was synthesized by reactive RF thermal plasma process from tungsten trioxide and nickel hydroxide blended micropowder. It was proven that most particles were alloyed because of co-condensation during rapid cooling from the conventional TEM sample of the nanoparticle. Part of them consisted of tungsten-rich part and nickel-rich part, which resulted to collision between them during flight. Occasionally, a core/shell-like particle was observed but it was ambiguous because of the limitation of plan-view analysis. The cross-sectional TEM sample of nanoparticle was prepared by impregnation and FIB milling procedure, and a sound sample was obtained without any artifacts. The tungsten-cored nickel shell-structured bimetallic nanoparticle was confirmed by high-resolution TEM and STEM on the cross-sectional sample.

## Abbreviations

FIB: focused ion beam; RF: radio frequency; STEM: scanning transmission electron microscopy

## Competing interests

The authors declare that they have no competing interests.

## Authors’ contributions

SJ performed the preparation of cross-sectional TEM sample and drafted the manuscript. HN and GL synthesized W-Ni nanoparticle and analyzed the morphology of the nanoparticle. SHS conducted TEM analysis. HC designed the cross-sectional TEM sampling of nanoparticle and finalized the manuscript. All authors discussed the results and read and approved the final manuscript.
